# Population Pharmacokinetic Modeling of Intravenous Topiramate in Patients with Epilepsy or Migraine

**DOI:** 10.1002/jcph.70191

**Published:** 2026-04-16

**Authors:** Adeboye O. Bamgboye, Lisa D. Coles, Bovornpat Suriyapakorn, Usha Mishra, Robert L. Kriel, Ilo E. Leppik, James R. White, James C. Cloyd

**Affiliations:** ^1^ Department of Experimental and Clinical Pharmacology University of Minnesota Minneapolis MN USA; ^2^ Center for Orphan Drug Research, College of Pharmacy University of Minnesota Minneapolis MN USA; ^3^ Department of Neurology University of Minnesota Minneapolis MN USA; ^4^ Minnesota Epilepsy Group University of Minnesota Roseville MN USA

**Keywords:** enzyme inducers, epilepsy, migraine, population pharmacokinetics, topiramate

## Abstract

Topiramate (TPM) is approved for seizures and migraine prophylaxis and is used off‐label for several neuropsychiatric conditions. The available dosage forms, including tablets and sprinkle capsules, are unsuitable for patients who may be unable to take medicine orally. The resulting potential treatment interruption could have untoward consequences and underscore the importance of developing a parenteral formulation. In this study, we developed a population pharmacokinetic model of a novel intravenous TPM formulation using data from a study in patients with epilepsy or migraine who received a single intravenous dose of stable‐isotope‐labeled TPM. In total, 246 TPM concentrations from 20 adult patients were included for model development. A three‐compartment pharmacokinetic model with linear elimination fit the concentration–time data best. Simulations for various loading doses in patients with and without enzyme‐inducing comedications were performed. The final estimates (95% confidence interval [CI]) for CL (L/h), V1 (L), and the peripheral volumes, V2 and V3, for a 70 kg person were 1.31 (1.01–1.53), 9.84 (8.49–11.0), 39.1 (36.5–41.8), and 9.01 (6.41–44.3), respectively. The use of enzyme‐inducing co‐medication was the only significant covariate, associated with a 63% increase in clearance. Goodness‐of‐fit plots and visual predictive checks indicate satisfactory model performance and prediction. Pharmacokinetic simulations indicated that IV TPM loading doses do not require adjustment when used as a replacement for interrupted oral therapy. This population pharmacokinetic model for intravenous topiramate can inform dosing decisions for patients with epilepsy when used as either initiation or bridging therapy.

## Introduction

Topiramate (TPM) is approved for seizures and migraine prophylaxis and is used off‐label for a growing list of neuropsychiatric conditions.^1^, ^2^ Currently approved TPM formulations include 25 to 200 mg tablets and sprinkle capsules. However, patients who cannot take oral TPM face potential treatment interruptions with untoward consequences. Development of an intravenous topiramate formulation is underway, and some studies have been published reporting the safety and pharmacokinetics in healthy individuals and patients.^3^, ^4^, ^5^

Topiramate pharmacology has been extensively studied. Mechanisms of action include blocking voltage‐gated Na^+^ ^6^ and Ca^2+^ channels.^7^, ^8^ enhancing GABA‐mediated transmission,^9^, ^10^ glutamate‐mediated transmission,^11^, ^12^ and inhibiting the carbonic anhydrase enzyme.^13^, ^14^, ^15^ However, the role of these mechanisms in terms of TPM's anti‐seizure effect is not well understood.

TPM exhibits linear pharmacokinetics at clinical doses, rapid absorption, and a half‐life of 20–30 h.^2^ ^,3,^ ^16^ Approximately 70%–80% of an administered dose is excreted unchanged in the urine.^17^ Several population pharmacokinetic models of oral TPM have been developed to characterize its pharmacokinetics and identify covariates that influence the pharmacokinetic parameters.^18^, ^19^, ^20^, ^21^ Although one study characterized the population pharmacokinetics of intravenous topiramate in healthy individuals,^21^ to our knowledge, there is no published population pharmacokinetic model of intravenous TPM in patients with epilepsy or migraine.

In this study, we developed the first population pharmacokinetic model of a novel, intravenous TPM formulation using data from a pilot phase I study in patients receiving a single intravenous dose of stable‐isotope‐labeled TPM.^4^ A portion of those patients were taking enzyme‐inducing comedications. The stable‐isotope‐labeled TPM formulation includes a ^13^C‐labeled (nonradioactive) isotope, which allows for safe and rigorous characterization of the pharmacokinetics of IV TPM without the need for washout or interruption of oral TPM therapy in these patients.^22^ The objectives of this study were to: (i) estimate the population pharmacokinetic parameters of IV TPM and their interindividual variability, (ii) identify potential covariates, including enzyme‐inducing comedications, influencing the estimated parameters, and (iii) simulate various loading doses for patients with and without inducing comedications to inform dosing of IV TPM.

## Methods

### Study Population

Twenty adult patients receiving maintenance oral TPM therapy for epilepsy or migraine, and administered a single 25mg intravenous dose of stable isotope‐labeled IV TPM, were included in this analysis. Seven (35%) were receiving enzyme‐inducing comedications. Of these, four were on carbamazepine, one on phenytoin, one on oxcarbazepine, and one was taking both carbamazepine and phenytoin. Individuals who were pregnant or breastfeeding were excluded. A history of intolerance to intravenous medication administration and known hypersensitivity to TPM were additional exclusion criteria for the study. Information about age, race, sex, renal function, and medical history was collected at the screening visit. Renal function was estimated using the Cockcroft–Gault equation (CrCL).^23^

### Study Drug Formulation and Measurement

The study drug was formulated as a 10 mg/mL solution dissolved in a 10% sulfobutyl cyclodextrin (Captisol, Ligand Technologies, La Jolla, CA, USA). Plasma concentrations of the stable isotope labeled IV TPM and oral TPM were quantified using a validated LC‐MS method with electrospray ionization and selected ion monitoring, utilizing distinct m/z transitions (m/z 338, m/z 344, and m/z 350 for oral TPM, stable isotope labeled IV TPM, and internal standard, respectively) to enable separate analyte quantification. A full description of the analytical method and study drug formulation is provided in a previous publication.^4^

### Study Design

The study was conducted under IND #78993 and approved by the University of Minnesota Institutional Review Board. All participants provided written informed consent.

A 25 mg dose of stable‐isotope‐labeled IV TPM was administered to patients along with their usual morning oral TPM dose. The infusion was administered over 10 min under monitoring conditions. Blood samples were drawn at predose, 5, 15, and 30 min, and at 1, 2, 4, 6, 12, 24, 48, 72, and 96 h after administration. Measurements of sodium, potassium, glucose, serum creatinine, urea nitrogen, hemoglobin, liver enzymes, and total plasma protein were done prior to IV administration. A full description of the study design is provided in a previous publication.^4^

### Population Pharmacokinetic (PopPK) Model Development

The PopPK model was developed using nonlinear mixed‐effects modelling software NONMEM (version 7.5.1; ICON Development Solutions, Ellicott City, MD, USA) with a Finch Studio interface (Enhanced Pharmacodynamics, LLC). The first‐order conditional estimation with interaction (FOCEI) was used for the estimation of parameters. Model selection was based on a significant change in the objective function value (OFV), Akaike information criterion (AIC), visual inspection of goodness‐of‐fit (GOF) plots, and visual predictive check. One‐, two‐, and three‐compartment pharmacokinetic (PK) models with linear elimination were tested. The models were parameterized using volume and clearance terms. The base model was designed to incorporate the effect of weight with a fixed allometric exponent of 0.75 for clearances and 1 for volumes to balance model complexity with physiological plausibility. The inter‐individual variability (IIV) was estimated using an exponential random effect model, consistent with standard PopPK modeling practice and ensuring parameter positivity, as described by the following equation:

$$\theta_{i}=\theta_{\mathrm{TV}}\times \exp\left(\eta i\right)$$

where ϴ_i_ is the individual PK parameter estimate, ϴ_TV_ is the typical value of the PK parameter, and ηi is the interindividual random effect with a mean of 0 and a variance of ⍵^2.^ The estimates of the IIV are presented as coefficients of variation expressed as a percentage (%CV) calculated using the equation below:

$$\%\mathrm{CV}(\mathrm{IIV})=\sqrt{\{e^{\omega^{2}}-1\}}$$

For the residual error model, we tested additive, proportional, and combined residual error models. The base model was selected based on the likelihood ratio test (LRT) of the change in the OFV. Covariate analysis was conducted using a stepwise method based on changes in OFV, evaluated with an LRT, to assess factors influencing TPM PK. The covariates tested included age, sex, inducer status, and creatinine clearance. These were added to the base model using a forward inclusion criterion of *P* <.05 (χ^2^, df = 1, **Δ**OFV of at least 3.84) and a backward elimination criterion of *P* <.01(χ^2^, df = 1, **Δ**OFV of at least 6.63), respectively.

### Model Evaluation

Model adequacy was assessed by successful minimization, significant changes in the objective function value (OFV) and Akaike information criterion (AIC), visual inspection of goodness‐of‐fit (GOF) plots and visual predictive check (1000 replicates of the original study dataset), precision of parameter estimates, and bootstrap resampling (n = 1000).

### Model Simulation

We also performed loading dose simulations in a reference individual (70 kg) to evaluate concentration‐time profiles across alternative loading doses. Although the FDA‐approved doses are not weight‐based, the loading dose simulation was conducted using a weight‐based dosing (mg/kg) to align with the population PK model structure, which incorporates weight as a covariate on clearance and volume of distribution.

## Results

### Patient Characteristics

**Figure 1:**
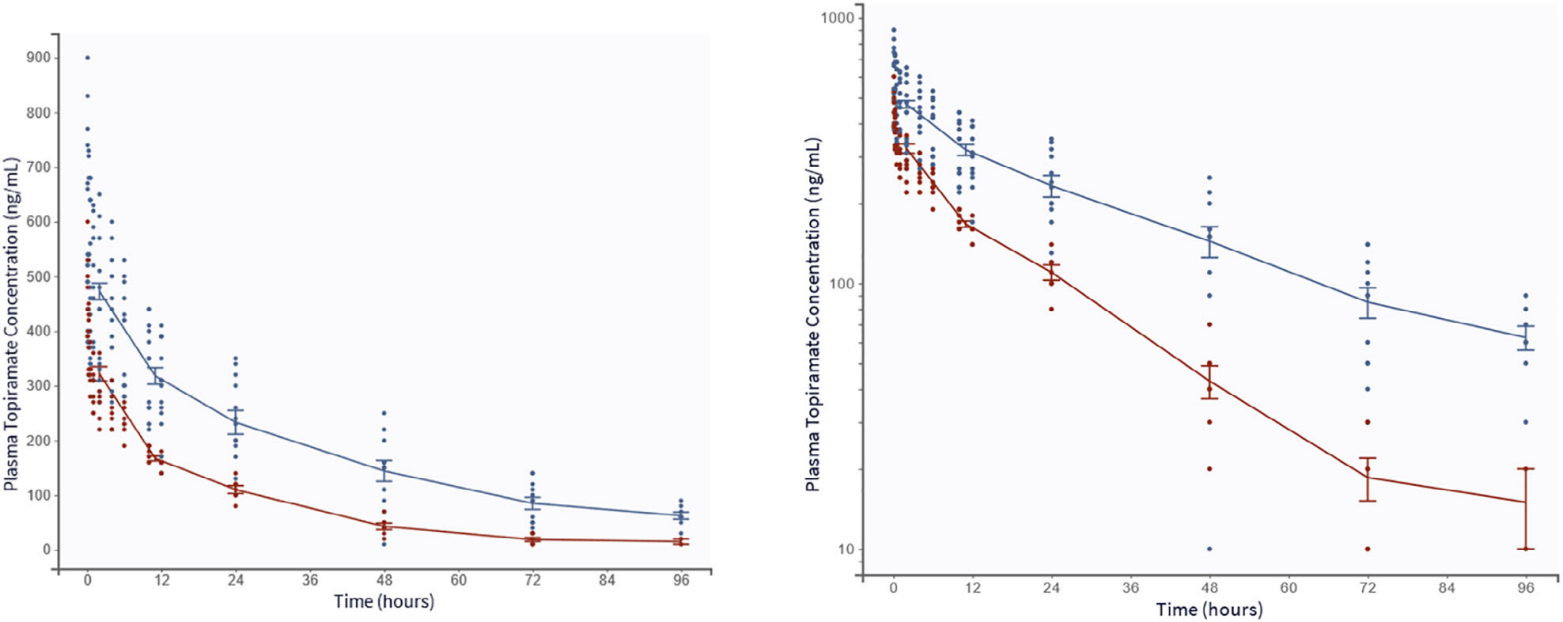
Mean plasma concentration–time profiles following a single 25mg intravenous dose of topiramate, shown on (a), linear and (b) semi‐logarithmic scales. The blue line represents the profile for individuals on monotherapy. The red line represents the profile for patients receiving enzyme‐inducing comedications. Points represent observations. Error bars represent standard errors.

A total of 246 PK observations (unique stable‐isotope‐labeled TPM concentrations) from 20 patients (13 females, 7 males, all Caucasian) were included in the analysis. The mean (SD) age for the patients was 39.8 (12.1) years, and the median (range) weight was 85.2 (54.5–150.3) kg. The median creatinine clearance was 107.6 mL/min (range: 46–206). The baseline characteristics of the studied population are summarized in Table 1. The concentration–time profiles (0–96 h) following a 25 mg intravenous dose stratified by inducer status are shown in Figure 1.

**Table 1:** Demographic and Baseline Characteristics

Parameters	Patients
n	20

Age(years)	
*Mean (SD)*	39.8 (12.1)
*Median(range)*	41.5 (26–74)

Height(cm)	
*Mean (SD)*	169 (8.36)
*Median(range)*	170 (155–182)

Weight (Kg)	
*Mean (SD)*	92.3 (26.6)
*Median(range)*	85.2 (54.5–150.3)

Sex, n (%)	
*Female*	13 (65)
*Male*	7 (35)

Race, n (%)	
*White*	20 (100)

Creatinine clearance (mL/min)	
*Mean (SD)*	106.7 (34.69)
*Median(range)*	102.5 (46–206)

Inducer comedications, n (%)	
*Received enzyme inducer*	7 (35)
*No enzyme inducer*	13 (65)

### Population PK Model

The final model was a three‐compartment model with linear elimination and an exponential IIV model for central clearance and volume. The final population PK parameters are presented in Table 2. Correlation parameters between CL, V1, and V2, and between V1 and V2, improved the model fit and were subsequently included. A proportional error model best described the residual unexplained variability. Results of the structural model selection and empirical Bayes estimate reliability assessment are presented in Tables S1 and S2, respectively. During the forward inclusion, Inducer on CL was the only significant covariate (ΔOFV > 3.84). Backward elimination resulted in a significant increase in OFV (ΔOFV > 6.63); therefore, the final model included inducer status as a predictor of CL. The results of the covariate analysis are summarized in Table S3. The quantitative relationships between model parameters and covariates are presented below:

$$\mathrm{CL}\left(L/h\right)=1.31\times {\left(\mathrm{WT}/70\right)}^{0.75}\times 1.63^{\mathrm{Inducer}}$$

$$V1\left(L\right)=15.6\times \left(\mathrm{WT}/70\right)$$

**Table 2:** Parameter Estimates of the Final Pharmacokinetic Model

	Final model		Bootstrap model (n = 1000)		
Parameter	Fixed effects estimate	RSE (%)	Bootstrap median	95% CI	Shrinkage (%)
CL (L/h)	1.31 × (WT/70)^0.75^	9.51	1.28	1.01–1.53	–
V1 (L)	9.84 × (WT/70)	8.7	9.83	8.49–11.0	–
Q2 (L/h)	197 × (WT/70)^0.75^	6.73	199	181–223	–
V2 (L)	39.1 × (WT/70)	5.1	38.9	36.5–41.8	–
Q3 (L/h)	0.6 × (WT/70)^0.75^	41.7	0.6	0.4–1.2	–
V3 (L)	9.01 × (WT/70)	18.8	9.44	6.41–44.3	–
**Covariate**					
Inducer ∼ CL	1.63	16.3	1.7	1.12–2.30	–
***Interindividual variability***					
IIV on CL (CV%)	33.5	40	32.4	20.2–52.6	1.48
Correlation (ΩCL,V1)	0.06	51.7	0.05	0.01–0.12	–
IIV on V1 (CV%)	18.1	74.2	17.5	5.5–32.0	1.5
Correlation (ΩV1,V2)	0.03	52.8	0.02	0.005–0.05	–
Correlation (ΩCL,V2)	0.03	60.4	0.03	0.005–0.07	–
IIV on V2 (CV%)	19.3	37.6	17.5	11.9–24.1	4.21
***Residual variability***					
Proportional (%CV)	0.02(12.8)	13.7	0.02	0.01–0.02	6.28

CL, central clearance; CV, coefficient of variation %CV = √(e^ω2^‐1); IIV, interindividual variability; Q2, intercompartmental clearance of the first peripheral compartment; Q3, intercompartmental clearance of the second peripheral compartment; RSE, relative standard error; V1, central volume of distribution; V2, volume of distribution of the first peripheral compartment; V3, volume of distribution of the second peripheral compartment; WT, bodyweight.

### Model Evaluation

**Figure 2:**
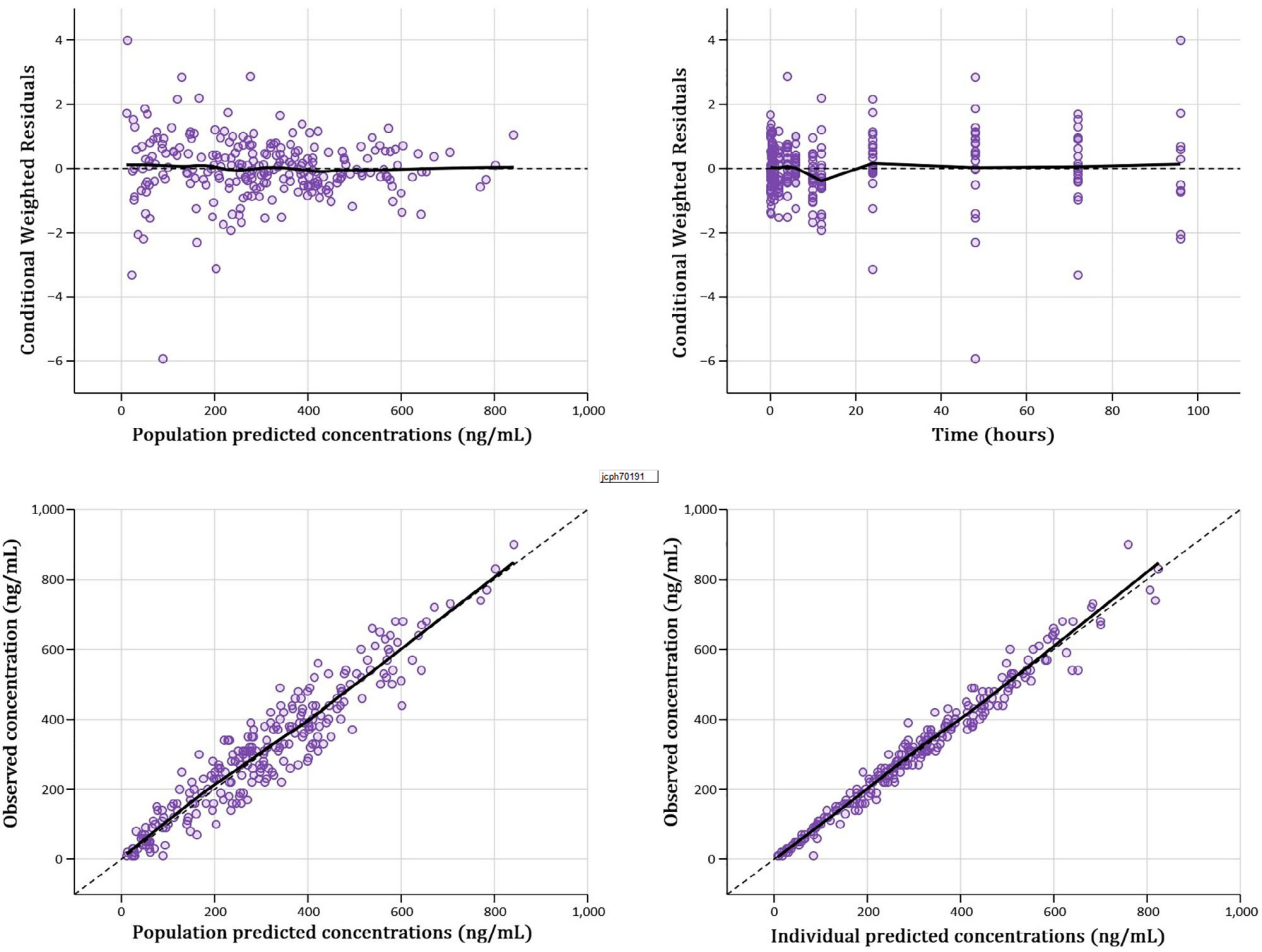
Goodness‐of‐fit plots of the final model. Conditional weighted residuals (CWRES) versus population predictions plot (upper left), CWRES versus time plot (upper right), individual observations (DV) versus population predicted concentrations (PRED) plot (lower left), and DV versus individual predicted concentrations (IPRED) plot (lower right). The dashed black lines indicate the reference lines, and the solid black lines show the smoothed trend in the data.

**Figure 3:**
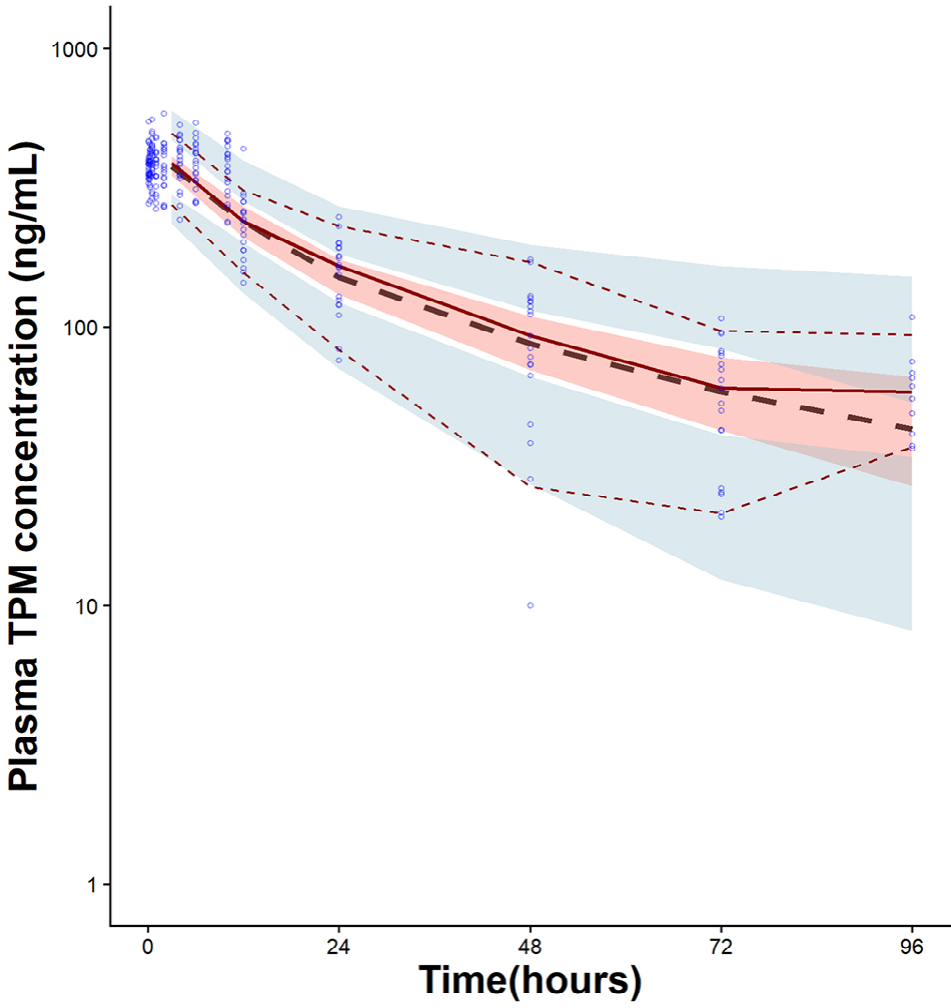
Prediction corrected visual predictive check (pcVPC) of the final population PK model based on 1000 simulations after a single 25 mg intravenous dose of TPM. Open blue circles represent observed data. The solid red line represents the median of observed concentrations. The dashed black line represents the median of the simulated concentrations. The upper and lower dashed red lines represent the 95th and 5th percentiles of observed data, respectively. The shaded pink area represents the confidence interval around the simulated median. The shaded blue areas represent the confidence intervals for the 5th and 95th percentiles of the simulated data.

The GOF plots are shown in Figure 2. Visual inspection of the GOF plots indicates that the final model adequately captures the data's trend. The observations (DV) versus population predictions (PRED) and individual predictions (IPRED) plots show a symmetrical distribution around the diagonal line, indicating good model fit. The CWRES versus PRED and Time plots show that a majority of residuals were within two standard deviations and evenly distributed around the line of identity, indicating proper specification of the structural and residual models. The means and 95% confidence intervals of the final PK parameters are shown in Table 2. Bootstrap resampling with 1000 replicates was implemented without stratification. A total of 925 bootstrap runs (92.5%) converged successfully. The medians of the PK parameters from bootstrap analysis were similar to the model estimates, indicating robustness of the parameter estimates. The prediction‐corrected VPC plot (Figure 3) shows that the model adequately captures the median observed trend and variability in the dataset. The stratified VPC (Figure S1) indicates that the final model more closely captures the median trend in the non‐inducer group than in the inducer group, potentially due to greater variability in the inducer group.

### Model Simulation

**Figure 4:**
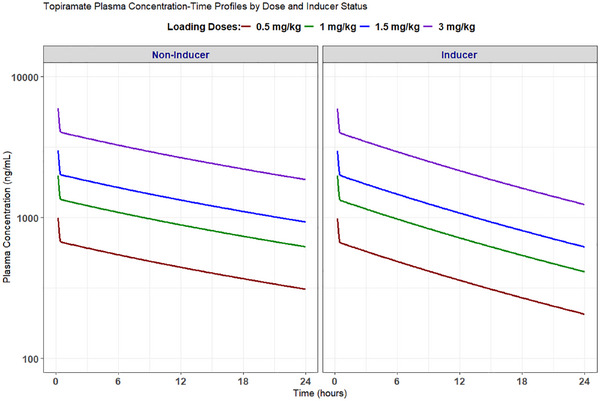
Simulated plasma concentration–time profiles following various loading doses of intravenous TPM in a 70 kg individual with and without concurrent enzyme inducers. Doses are represented as follows: 3 mg/kg (purple), 1.5 mg/kg (blue), 1 mg/kg (green), and 0.5 mg/kg (red).

The loading dose simulations showed dose‐proportional increases in C_max_ across doses irrespective of enzyme induction status (Figure 4).

## Discussion

We report the first population pharmacokinetic analysis of intravenous topiramate in patients with epilepsy or migraine. In this study, we found that a three‐compartment pharmacokinetic model fit the TPM concentration‐time data best for patients with epilepsy or migraines. We also found that enzyme‐inducing comedications significantly increased TPM clearance in this population. In contrast to many other population pharmacokinetic models of oral topiramate that were described by a one‐ or two‐compartment model, a three‐compartment model fit our data best. This result indicates that TPM's distribution is more complex than previously thought. While many studies with oral topiramate used sparse steady‐state concentrations for modeling, our study used rich sampling, particularly at early time points. We believe this approach enabled better characterization of TPM PK, particularly early‐phase distribution kinetics, which is critical to guide IV TPM dosing. Notably, another PopPK study of IV and oral TPM with rich sampling in healthy volunteers supported a two‐compartmental structural model.^21^ In that study, the authors concurrently modeled oral and IV TPM concentration data. In our model, topiramate central clearance for an adult (70kg) not on inducing comedications was estimated to be 1.31 L/h, which is consistent with clearance estimates (1.16–1.47 L/h) from previous studies of oral TPM in healthy volunteers and patients with epilepsy.^3^, ^17^, ^19^

We found that TPM clearance increased by 63% (coefficient for inducer effect = 1.63) in patients receiving enzyme inducers. This finding is consistent with previously published studies reporting increases of 15%–94%.^17^, ^20^, ^24^ A direct consequence of the increased clearance is the need to adjust doses for patients receiving TPM with enzyme‐inducing comedications. While some studies modeled the influence of single enzyme‐inducing comedications,^17^, ^19^ data limitations and a need to avoid overparameterization led to a simplified binary covariate approach for modeling the effect of enzyme inducers on clearance. Our PopPK simulations show that changes to loading doses are not warranted in this population, as loading doses typically depend on volume of distribution, which is not affected by enzyme‐inducers (Figure 4).

The observed increase in clearance is notable and raises important questions about the mechanisms that may contribute to it. Given that TPM is primarily excreted renally, the considerable difference in CL suggests that both hepatic and renal CL are influenced either directly or indirectly by enzyme‐inducing medications. As an example, it is plausible that enzyme‐inducing medications interact with renal tubular active transporters that play a role in the renal excretion of TPM. Further studies are needed to rigorously describe the extrahepatic mechanisms involved in TPM clearance when co‐administered with enzyme inducers.

We also investigated the influence of other covariates on IV TPM clearance. Age, sex, and creatinine clearance (CrCL) were not significant covariates for clearance. While a previous study found clearance to increase with age,^17^ no such relationship was identified in our study, possibly reflecting differences in age distributions between studies. Similarly, renal function has been reported to influence TPM clearance in several published studies.^17^, ^18^, ^19^ We did not include race in the covariate modeling because we lacked data to reliably estimate its effect. One study^21^ included race as a covariate, but did not find a relationship between clearance and race after accounting for renal function.^21^ At present, there is no evidence to suggest that TPM clearance differs across racial groups; however, further research is needed.

The standard errors of the final model were acceptable, indicating reliable estimation of the parameters. The interindividual variability in clearance was 33.5%, which is consistent with estimates from previous studies,^17^, ^20^, ^25^ demonstrating considerable variability in drug distribution among individuals. Bootstrap and VPC results indicate that the model exhibits stability and satisfactory reproducibility.

It is worth mentioning some limitations of our study. Condensing the enzyme‐inducer effect into a single covariate assumes that the changes in TPM clearance as a function of enzyme‐inducing comedications are uniform across commonly co‐administered medications such as carbamazepine and phenytoin. Results from previous studies suggest that the increase in TPM clearance may depend on the specific inducing co‐medication administered.^17^, ^19^ While previous studies have shown that renal function influences TPM clearance,^17^, ^18^ there was no significant relationship between CrCL and TPM clearance in our model. This lack of an effect could be due to the near‐normal CrCL values of the patient population. As a result, our model may not generalize well to patient populations with renal impairment. Future studies evaluating renal function, including CrCL as a covariate for IV TPM clearance in larger patient cohorts, are warranted to improve the clinical utility of the model. The modest sample size may reduce the ability to fully characterize interindividual variability and covariate effects. The bootstrap analysis was not stratified by inducer status, which may limit the ability to fully account for subgroup‐specific variability.

## Conclusion

In conclusion, we successfully developed a population model to describe intravenous topiramate pharmacokinetics in patients with epilepsy or migraine. The model adequately captured the data trend, as indicated by the standard error estimates of the parameters and by visual inspection of the diagnostic plots. Inducer status was the only significant covariate that influenced topiramate clearance. IV TPM clearance increases by 63% for individuals receiving concomitant enzyme inducers. Additional studies, including patients with a broader range of renal function, may improve the precision and clinical utility of the model. Overall, our findings translate model‐based insights into a framework for informing future IV TPM dosing strategies.

## Author Contributions

Adeboye O. Bamgboye, Lisa D. Coles, Ilo E. Leppik, Robert L. Kriel, James R. White, and James C. Cloyd wrote the manuscript. Lisa D. Coles, James C. Cloyd, and Adeboye O. Bamgboye designed the study. Usha Mishra, James C. Cloyd, and Ilo E. Leppik performed the research. Adeboye O. Bamgboye, Bovornpat Suriyapakorn, Lisa D. Coles, and James C. Cloyd analyzed the data.

## Conflicts of Interest

James C. Cloyd is entitled to royalties under a licensing agreement pertaining to injectable topiramate between Ligand Pharmaceuticals, Inc., and the University of Minnesota. This relationship has been reviewed and managed by the University of Minnesota in accordance with its conflict of interest policies.

## Funding

The authors received no funding for this work.

## Supporting information



Supplementary Information.

## Data Availability

The data that support the findings of this study are available on request from the corresponding author. The data are not publicly available due to privacy or ethical restrictions.
