# Supplementary material for: Population Pharmacokinetic Modeling of Intravenous Topiramate in Patients with Epilepsy or Migraine
Source: J Clin Pharmacol. 2026 Apr 16;66:e70191. doi: 10.1002/jcph.70191 (PMC13084296; doi:10.1002/jcph.70191)
Supplement: Supplementary file 1 — Supplementary Information. [file JCPH-66-0-s001.docx]

**Supplementary materials**

**Table S1**. Quantitative model selection criteria comparison

| **Model structure** | **OFV** | **ΔOFV vs**  **1-compartment** | **AIC** | **BIC** | **Number of parameters** | **Comments** |
| --- | --- | --- | --- | --- | --- | --- |
| 1-compartment | 2626.2 | **–** | 2638.2 | 2659.2 | 5 | Baseline |
| 2-compartment | 2153.5 | -472.7 | 2169.5 | 2197.5 | 7 | Considerable improvement |
| 3-compartment | 2113.43 | -512.8 | 2133.4 | 2168.5 | 9 | Significant additional improvement |
| OFV, AIC, and BIC all favor the 3-compartment model | | | | | | |

**OFV**: objective function value, **AIC**: Akaike information criterion, **BIC**: Bayesian information criterion

**Table S2**. Random-effects diagnostics supporting EBE reliability

| **Model** | **η-shrinkage(%)**  **on CL** | **η-shrinkage(%)**  **on V1** | **η-shrinkage(%)**  **on V2** | **ε-shrinkage(%)** |
| --- | --- | --- | --- | --- |
| 1-compartment | 4.46 | 4.46 | - | 2.28 |
| 2-compartment | 1.26 | 6.14 | - | 5.33 |
| 3-compartment | 1.48 | 1.50 | 4.21 | 7.55 |

**ETA(η):** interindividual random effects, **EPSILON(ε):** residual unexplained variability, **EBE:** empirical bayes estimate

**Table S3**. Covariate Search

| **Model** | **Description** | **OFV** | **ΔOFV** | **Comment** |
| --- | --- | --- | --- | --- |
| 1 | 3CMT, WT on param, prop error | 2081.86 | – | 3-compartment model, weight on clearances and volumes, proportional error model, diagonal omega matrix |
| 2 | 3CMT, WT on param, prop error, IIV on CL, V1 and V2 | 2051.56 | 30.3 | 3-compartment model, weight on clearances and volume, proportional error model, IIV on CL, V1 and V2 |
| Forward Inclusion **[Threshold is ΔOFV > 3.84]** | | | | |
| 3 | Age on CL and V1 | 2050.61 | -0.95 | Adding age as a covariate on CL and V1 |
| 4 | Height on CL and V1 | 2050.03 | -1.53 | Adding height as a covariate on CL and V1 |
| 5 | Inducer on CL | 2043.81 | -7.75 | Adding inducer on CL |
| 6 | Inducer and CrCL on CL | 2041.97 | -1.84 | Adding inducer and creatinine clearance as a covariate on CL |
| Backward Elimination **[Threshold is ΔOFV > 6.63]** | | | | |
| 7 | Removing inducer on CL | 2051.56 | 7.75 | Retain inducer as a covariate on CL |
| **End of backward step: Include inducer on CL** | | | | |

OFV: objective function value, CMT: compartment, WT: body weight , CL: central clearance, V1: central volume of distribution, CrCL: creatinine clearance, IIV: interindividual variability


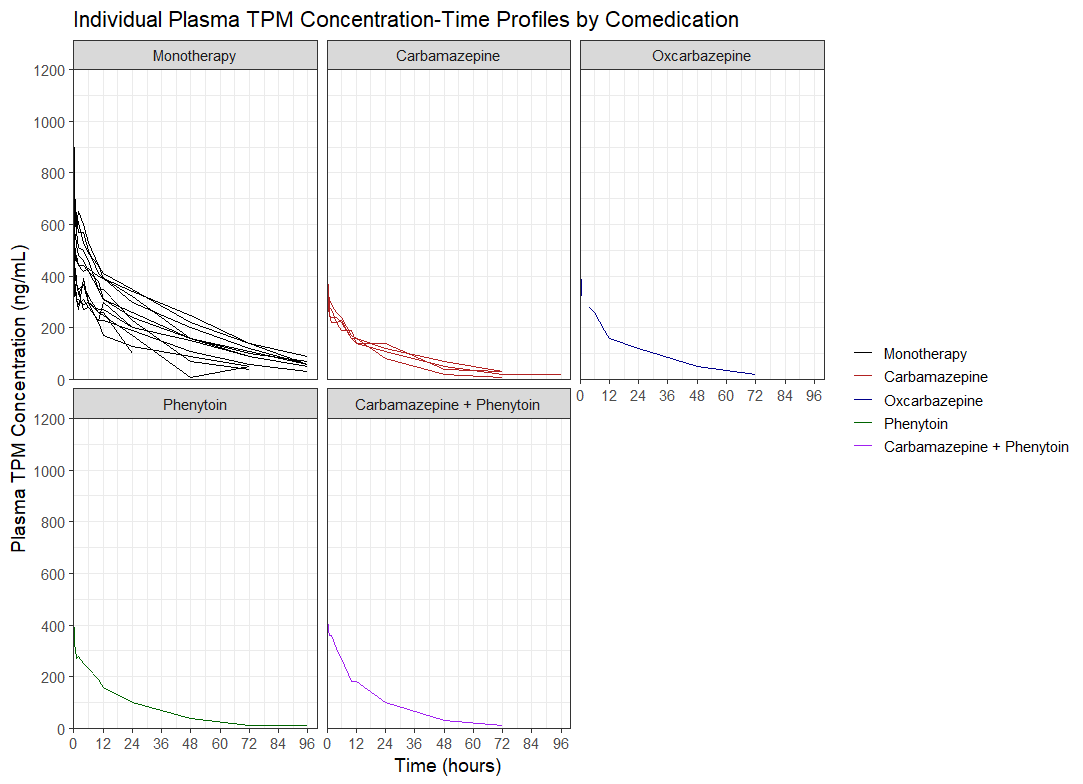


**Figure S1**. Individual plasma TPM concentration-time profiles by comedication

TPM : topiramate
